# Evaluating the efficacy of different volume resuscitation strategies in acute pancreatitis patients: a systematic review and meta-analysis

**DOI:** 10.1186/s12876-024-03205-y

**Published:** 2024-03-25

**Authors:** Roopa Kumari, FNU Sadarat, Sindhu Luhana, Om Parkash, Abhi Chand Lohana, Zubair Rahaman, Hong Yu Wang, Yaqub N Mohammed, Sanjay Kirshan Kumar, Subhash Chander

**Affiliations:** 1 Department of Medicine, Icahn School of Medicine at Mount Sinai, 1 Gustave L. Levy PI, New York, NY USA; 2 Department of Medicine, University of Buffalo, New York, USA; 3 Department of Medicine, Montefiore Medical Center, Weikfield, NY USA; 4 Department of Medicine, WVU Camden Clark Medical Center, West, VA USA; 5 Department of Medicine, Western Michigan University, Pontiac, USA; 6 Department of Medicine, Bahria University Health Sciences Karachi, Karachi, Pakistan

**Keywords:** Acute pancreatitis, Fluid resuscitation, Clinical outcomes, Mortality, Type of Fluids

## Abstract

**Introduction:**

Acute pancreatitis poses a significant health risk due to the potential for pancreatic necrosis and multi-organ failure. Fluid resuscitation has demonstrated positive effects; however, consensus on the ideal intravenous fluid type and infusion rate for optimal patient outcomes remains elusive.

**Methods:**

A comprehensive literature search was conducted using PubMed, Embase, the Cochrane Library, Scopus, and Google Scholar for studies published between 2005 and January 2023. Reference lists of potential studies were manually searched to identify additional relevant articles. Randomized controlled trials and retrospective studies comparing high (≥ 20 ml/kg/h), moderate (≥ 10 to < 20 ml/kg/h), and low (5 to < 10 ml/kg/h) fluid therapy in acute pancreatitis were considered.

**Results:**

Twelve studies met our inclusion criteria. Results indicated improved clinical outcomes with low versus moderate fluid therapy (OR = 0.73; 95% CI [0.13, 4.03]; *p* = 0.71) but higher mortality rates with low compared to moderate (OR = 0.80; 95% CI [0.37, 1.70]; *p* = 0.55), moderate compared to high (OR = 0.58; 95% CI [0.41, 0.81], *p* = 0.001), and low compared to high fluids (OR = 0.42; 95% CI [0.16, 1.10]; *P* = 0.08). Systematic complications improved with moderate versus low fluid therapy (OR = 1.22; 95% CI [0.84, 1.78]; *p* = 0.29), but no difference was found between moderate and high fluid therapy (OR = 0.59; 95% CI [0.41, 0.86]; *p* = 0.006).

**Discussion:**

This meta-analysis revealed differences in the clinical outcomes of patients with AP receiving low, moderate, and high fluid resuscitation. Low fluid infusion demonstrated better clinical outcomes but higher mortality, systemic complications, and SIRS persistence than moderate or high fluid therapy. Early fluid administration yielded better results than rapid fluid resuscitation.

## Introduction

Acute pancreatitis (AP) is an acute inflammation of the pancreas and one of the leading global causes of hospitalization for gastrointestinal complications [1]. Heckler et al. indicated that approximately 20% of AP cases usually progress to severe pancreatitis, leading to pancreatic necrosis and multi-organ failure; hence, the reason for the continued increase in mortality rate is currently estimated to be 40% [2]. For instance, pancreatic necrosis is mainly characterized by fluid loss due to hypoperfusion, splanchnic vasoconstriction, and reduced blood flow into the pancreas [3]. Accordingly, reduced blood flow premediates compromised microcirculation within the pancreas, which plays a significant role in the development of necrotizing pancreatitis.

Over the years, fluid resuscitation or adequate fluid resuscitation has been labeled as the main management approach for the early onset of acute pancreatitis, coupled with early oral feeding and pain management [4, 5]. For instance, with its significant role in minimizing mortality, scholars have suggested that early fluid resuscitation seamlessly prevents and limits pancreatic necrosis, inhibits prevalent multi-organ failure, minimizes the systemic inflammatory response, and enhances microcirculation in the pancreas [6].

Despite fluid resuscitation showing phenomenal treatment outcomes, there are yet exorbitant uncertainties in choosing the most appropriate fluid type and volumetric rates that maximize patient outcomes when administered. Blood products, colloids such as albumin, gelatin solutions, hydroxyethyl starch, and crystalloids, including normal saline and Ringer’s lactate, are considered fluid types for achieving fluid resuscitation [7–9]. Generally balanced crystalloid such as Ringer’s lactate is considered ideal for aggressive fluid replacement therapy for critically ill patients including those with AP [10].

Notwithstanding, the rate of fluid resuscitation is the epitome of controversy since literature has witnessed the administration of either low, moderate, or high rates of fluid resuscitation, which contribute to different clinical outcomes. Proponents of high fluid therapy position that high-rate fluid resuscitation therapy significantly reduces mortality in patients with pancreatitis [11]. On the other hand, opponents of the latter argue that high-rate fluid therapy potentially causes fluid overload, further worsening or precipitating respiratory and cardiac failure [12]. Moreover, studies investigating the clinical outcomes of low-rate fluid resuscitation reported better clinical outcomes, including bolstered tissue perfusion, minimized mortality, systemic inflammatory response syndrome, and reduced hospital stay [5]. The discrepancy in infusion rates of intravenous resuscitation fluid therapy is also evident across some of the major guidelines of the international association. For instance, renowned guidelines, IAP/APA evidence-based guidelines for managing acute pancreatitis, and American College of Gastroenterology guidelines for managing acute pancreatitis also show contradictions concerning fluid therapy infusion rates [13, 14]. Similarly, systematic reviews have highlighted the uncertainties and discrepancies attributed to fluid resuscitation administration rates in patients with acute pancreatitis.

Owing to its perceived safety, wide and ready availability, low cost, and simplicity, intravenous fluid therapy in the treatment of acute pancreatitis, this study sought to address the knowledge gap pertaining to the lack of conclusive evidence for an informed fluid resuscitation infusion rate that maximizes clinical outcomes for patients with pancreatitis. In this context, the current systematic review and meta-analysis aimed to systematically collate and appraise evidence through outcome assessments on the efficacy of low, moderate, or high infusion rates of fluid resuscitation to inform on the most appropriate infusion choice for acute pancreatitis patients.

## Methodology

### Search criteria

The present systematic review and meta-analysis was performed according to the Cochrane Collaboration Search Strategy and Preferred Reporting Items for Systematic Reviews and Meta-Analyses (PRISMA) protocols [15, 16].

### Eligibility: inclusion and exclusion criteria

The PICOTS framework provided the selection criteria used in this study. The included studies were conducted for at least ten years (Table 1).

**Table 1:** PICOS frameworks applied in the study

POPULATION	Acute pancreatitis patients
INTERVENTION	Fluid resuscitation infusion rates & types (low vs. moderate vs. high & early/slow vs. rapid) in acute pancreatitis
COMPARISON	Comparisons will be performed among studies reporting high vs. low fluid resuscitation, moderate vs. high fluid resuscitation, low vs. moderate resuscitation, and finally, early/slow vs. rapid fluid infusion.
OUTCOMES	The main outcomes of the present study include systemic anti-inflammatory response syndrome (SIRS), improved clinical outcomes, mortality incidences, and local complications including persistent organ failure.
TIMING	English language articles published from 2005 to January 2023
SETTING & DESIGN	RCTs, non-randomized controlled trials, retrospective cohorts, and prospective cohorts.

### Exclusion criteria

The following studies were excluded from the meta-analysis:

- Abstract, ongoing investigations, case studies, personal opinions, encyclopedias, and studies reporting outcomes irrelevant to the current topic.

### Search strategy

A detailed literature search was electronically performed by two investigators (DW and GH) on the following medical databases: PubMed, Embase, Cochrane Library, Scopus (Medline), and Google Scholar to identify eligible studies. The literature search was limited to studies reporting the outcomes of studies performed on humans, focusing on recent publications. The following keywords were used in the electronic databases: acute pancreatitis, fluid resuscitation, and fluid therapy. Additionally, a manual search was performed on the selected sources’ reference lists to identify potential studies.

### Study selection & data extraction

Two authors (SC and RK) independently selected eligible studies and extracted data from all articles selected for inclusion using a standard data extraction form. All citations were electronically retrieved from biomedical databases, after which they were scrutinized by the author (SC and RK), where studies that met the inclusion criteria were not included in the present study. A systematic approach was deployed during the study selection and data extraction. First, duplicates were excluded. Second, the authors critiqued the titles and abstracts of eligible studies to filter and eliminate studies inconsistent with the inclusion criteria. Third, the studies were subjected to full-text analysis to ascertain their consistency with inclusion requirements. Finally, the author resolved conflicts arising from the studies through dialogue.

Two authors (SC and RK) independently extracted data from eligible studies using standard data extraction forms. Data extracted from the studies included study name, country of origin, number of participants, comparisons, and their respective outcomes.

### Risk of bias & study methodological quality assessment

The reviewers deployed a tool developed by the Cochrane Collaboration to assess the risk of bias in the included studies based on the following seven key domains: random sequence generation, allocation concealment, blinding of participants and personnel, blinding of outcome assessment, incomplete outcome data, selective reporting, and other risk factors. Each of the bias domains was categorized as either “high,” “unclear,” or “low” risk based on the author’s judgment of the assessment criteria. The risk of bias assessment was performed independently; however, a senior reviewer was consulted in case of any differences between investigators.

Regarding the quality of evidence of each included study, the GRADE assessment criteria were used, and the overall quality of the studies was deemed low, moderate, or high, depending on the scores on the five domains: study limitation, consistency, directness, precision, and publication bias (Table 2).

**Table 2:** Summary & key characteristics of included studies

Study ID, Year of Publication	Study design	Country of origin	Participants	Rate of IV fluids	Intervention	Total IV fluids received	Outcomes
De-Madaria et al., 2022 [19]	RCT	India, Italy, Spain, and Mexico	249	20 ml/kg bolus followed by 3 ml/kg/hr. vs. 10 ml/kg bolus followed by 1.5 cc/kg/hr.	Lactated Ringer’s solution Moderate vs. Low Fluid resuscitation group	Moderate 8.3 lit. Low 6.6 L	***Aggressive vs. Moderate*** ICU Admissions: 6.6% vs. 1.6% Persistent any organ failure: 6.6% vs. 1.6% Moderately or severe pancreatitis: 22.1% vs. 17.3% Necrotizing pancreatitis: 13.9% vs. 7.1% SIRS at 72 h. 8.8% vs. 14.3 Renal Failure: 7.4% vs. 2.4% Death 3.3% vs. 0.8% Fluid overload: 20.5% vs. 6.3%
Buxbaum et al., 2017 [18]	RCT	USA	60	20 ml/kg bolus vs. 10 ml/kg	Lactated Ringer’s solution Moderate 20 ml/kg vs. low 10 ml/kg	7.6 L vs. 5.6 L	Aggressive hydration showed more frequent clinical improvement, SIRS, and less development of hemoconcentration.
Angsubhakorn et al., 2021 [20]	RCT	Thailand	46	20 ml/kg bolus vs. 10 ml/kg	Lactated Ringer’s solution Moderate 20 ml/kg vs. low 10 ml/kg	4886 vs. 3985	45.5% of 22 patients in aggressive hydration reported improved SIRS vs. 31.82% in standard hydration.
Cuéllar-Monterrubio et al., 2020 [22]	RCT	Mexico	88	30 ml/kg vs. 20 ml/kg	Hartmann’s solution High 30 ml/kg vs. moderate 20 ml/kg	8540 vs. 5130	Patient with non-aggressive hydration developed more AKI as compared to aggressive group (6.9% vs. 11.1%). There is no difference in respiratory complication, pancreatic necrosis, and clinical improvement.
Lan Li et al., 2019 [27]	Cohort study	China	912	> 3 ml/hr vs. <3 ml/hr	Normal saline or Ringer’s lactate solution Moderate vs. Low	NA	Patient with high fluid rate developed more AKI. (22.4% vs. 17.2%) Multiple organ failure: >3 ml/h (28.6% vs. 13.8%) ICU admission: >3 ml/kg/h (87.8% vs. 79.3%) IMV: > 3 ml/kg/h (30.6% vs. 20.7%) NPPV: >3 ml/kg/h (83.7% vs. 62.1%)
Ahmed A. Messallam et al., 2021 [23]	RCT	USA	310	NA	Any fluid (Normal/D5 saline, Ringer’s lactate solution, and others) High vs. moderate vs. low	< 2.8 L vs. 4.5 L vs. > 4.5 L	**Organ failure**: Aggressive group 16.5 Moderate 7.6 vs. Conservative 4.9% **Mortality**: 8.7% vs. 2.9% vs. 2.0% Respectively
Enrique de-Madaria 2011 [21]	Cohort	Spain	247	NA	Fluid type not mentioned. Low vs. Moderate vs. High	< 3.1 L vs. 3.1-to-4.1-liter vs. > 4.1 L	High fluids reported more organ failure, respiratory complication and renal failure as compared to low and moderate group.
Vikesh K Singh 2017 [26]	Cohort	Multiple Countries*	1010	NA	Any fluid Low vs. Moderate vs. Low	< 3200 vs. 3200 to 4300 vs. > 4300	Moderate group associate with lower local complications. No difference in mortality in all groups Aggressive group required more invasive interventions.
Takahiro Yamashita 2018 [25]	Cohort	Japan	1097	NA	Any fluid High vs. Low	3922 vs. 8706	**Mechanical ventilation** Low resuscitation 18.2% vs. High resuscitation 51.7% **Renal replacement therapy 75** High resuscitation 10.6% vs. Low resuscitation 22.3% **Mortality** High resuscitation 15.9% vs. Low resuscitation 10.3%
Bo Ye et al., 2018 [24]	RCT	China	179	NA	Any fluid Moderate vs. Low	Aggressive vs. Nonaggressive 4501 vs. 3316	Patients with aggressive fluids developed more AKI than non-aggressive fluids. 16.36% vs. 4.86% Aggressive group received more renal replacement therapy than non-aggressive group. 40.63% vs. 24.36%
Warndorf et al. [29] 2011	Retrospective study	United states of America	434	> 3 ml/hr vs. <3 ml/hr.	Any fluid Early vs. late	Early (< 3 ml/kg/h) vs. Late/rapid (s ≥ 3 ml/ kg/h).	Early resuscitation was associated with decreased SIRS, compared with late resuscitation, at 24 and 72 h as well as reduced organ failure at 72 h a lower rate of admission to the intensive-care unit, and a reduced length of hospital stay
Gardner et al. [28] 2009	Retrospective study	United States of America	45	> 3 ml/hr vs. <3 ml/hr.	Any fluid Early vs. late	NA	The ‘late resuscitation’ group experienced greater mortality than those in the ‘early resuscitation’ group (18 vs. 0%, *p* < 0.04) and demonstrated a trend toward greater rates of persistent organ.

### Statistical analysis

All statistical analyses were performed using the Cochrane Collaboration Review Manager software (RevMan: version 5.4.1). Differences in dichotomous variables were calculated using an odds ratio (OR) and respective 95% confidence intervals (CI); for continuous variables, funnel plots, and forest plots were automatically generated using RevMan software. Heterogeneity between the studies was statistically assessed using the Chi-squared test, with significance set to a *p*-value of 0.10, and the quantity of heterogeneity was measured using the I^2^ statistic. Inconsistency and the degree of heterogeneity were divided into four parts % to 40%, might not be necessary; 30–60%, moderate heterogeneity; 50–90%, substantial heterogeneity; 75–100%, and considerable heterogeneity. Two approaches were used in the present meta-analysis: the random-effects model approach to examine inter-study heterogeneity and the Mantel-Haeszel fixed-effects model when no inter-study heterogeneity was established. Otherwise, the Mantel-Haenszel random-effects model was deployed when the studies presented significant heterogeneity. Publication bias was evaluated by visually inspecting funnel plots using Egger’s test on the line of asymmetry [17].

## Results

### Literature search and selection results

**Fig. 1:**
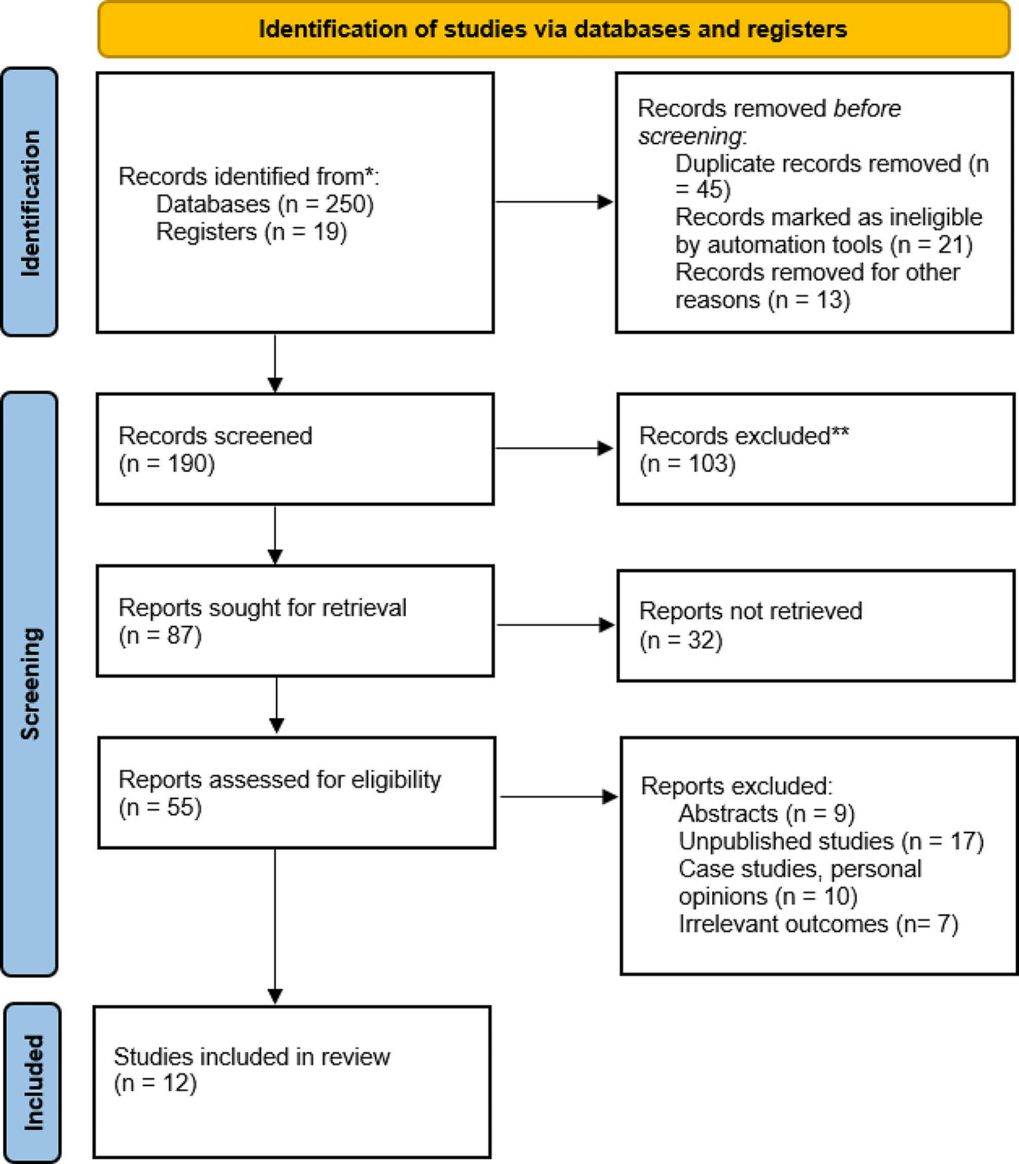
PRISMA flow diagram of the study selection process

In the initial literature review, 250 articles were identified in the databases, whereas 19 others were registered. Forty-five duplicates were excluded before screening. At the same time, automated tools marked 21 articles as ineligible, as 13 others were removed for other reasons. The remaining 190 articles were screened, leading to the exclusion of 103 records. Eighty-seven remaining studies were sought for retrieval, of which 32 still needed to be retrieved. Fifty-five studies remained and were assessed for eligibility: nine abstracts, 17 unpublished studies, 10 case studies, and seven irrelevant studies. Finally, only 12 studies that met the inclusion criteria were included (Fig. 1).

### Risk of bias of included studies

**Fig. 2:**
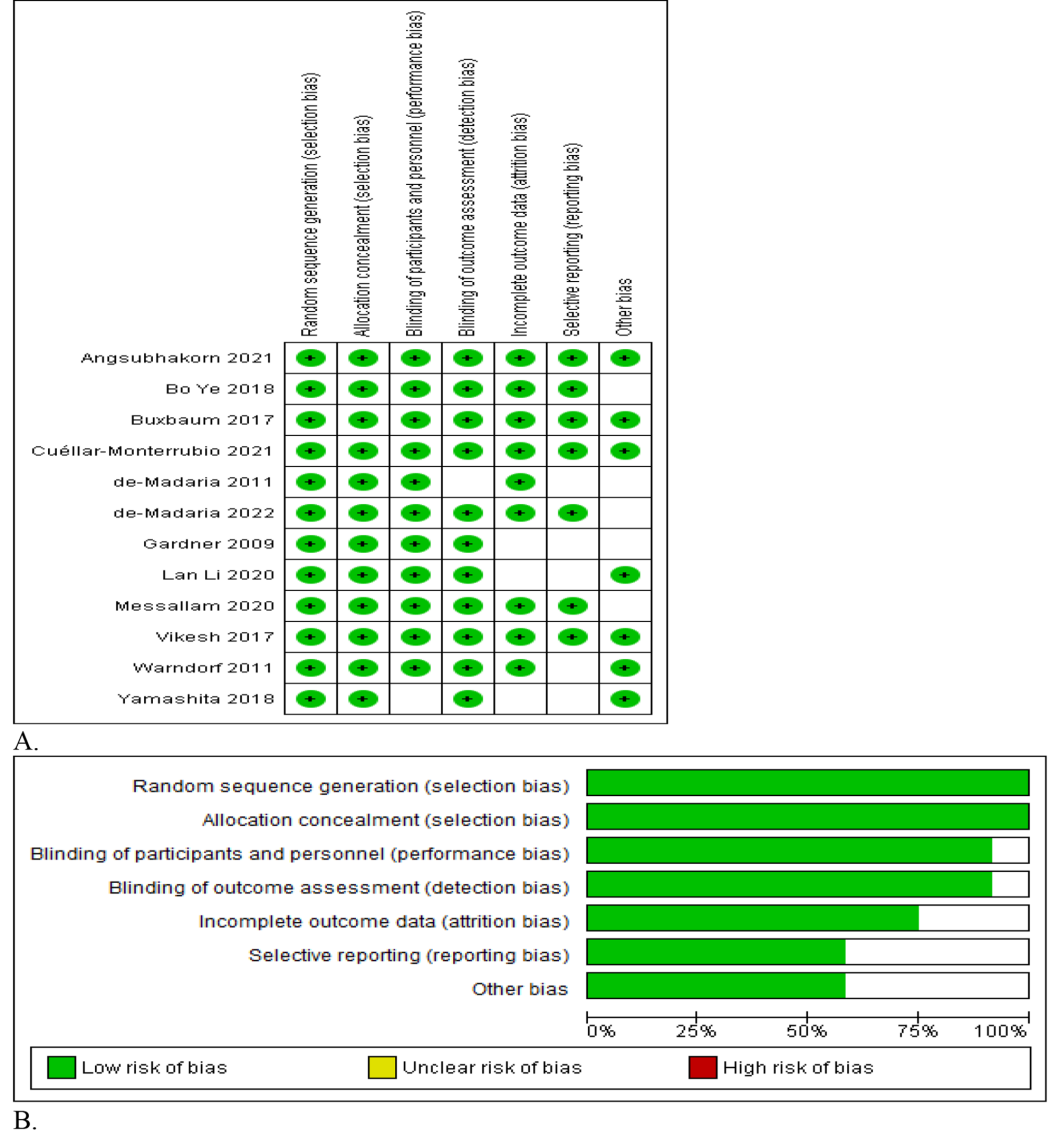
Risk of bias summary (**A**) and risk of bias graph (**B**)

The studies included in this review were assessed for their quality and risk of bias using Cochrane Collaboration’s risk of bias tool, as illustrated in (Fig. 2). Based on the risk of the assessment tool, all studies were classified as having high quality, except for four fair studies that showed an unclear risk of bias in the three criteria of bias assessment.

### Characteristics of included studies

Twelve studies (six RCTs and six cohort studies) [18–29] with 4,667 participants were included in this study. One study included patients with severe AP [28], two studies each with mild [18, 20] and moderately severe to severe AP [19, 24], while six included patients with different AP severity based on the Atlanta or bedside index for severity of acute pancreatitis classifications [21–23, 25, 27, 29]. The volumetric attributes of intravenous fluid resuscitation were evaluated based on the infusion rates (low vs. moderate vs. high fluid) in patients with acute pancreatitis. Among the included studies, despite the large population, there were variations, with the least registered per study being 45 participants [28] and the largest being 1097 participants [25]. All included studies, except three, provided comparative data on low (non-aggressive), moderate, and high (aggressive) fluid resuscitation volumes, apart from studies providing information based on early or late (rapid) fluid rates. The intervention fluid comprised Ringer’s lactate in three [18–20], Hartmann’s solution in one [22], saline or Ringer’s lactate in one [27], and any fluid type in six [23–26, 28, 29]. One study did not mention the type of intervention fluid used [21]. Furthermore, four studies originated from the United States of America [18, 23, 28, 29], one from Thailand [20], two studies originated from China [24, 27], one study each from Spain [21] and Mexico [22], and the rest were multinational studies [19, 26] (Table 2).

### Outcomes and results: comparison by infusion volume rates

#### Improvement of clinical outcomes

Three RCTs among the included studies [18, 20, 22], with a sample size of 184 participants, reported improved clinical performance regarding the administration of low, moderate, or high intravenous fluids, among which Buxbaum et al. [18] and Angsubhakorn et al. [20] compared low versus moderate intravenous fluid infusion. The heterogeneity test result (I² = 97%) was significantly high, implying that the studies showed substantial differences. Hence, a random effects model was applied to the statistical summation of the overall results.

**Fig. 3:**
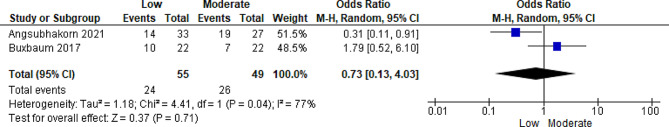
Forest plot of comparison: clinical outcome improvements

In a meta-analysis that included 104 participants, low fluid resuscitation was associated with improved clinical outcomes (OR = 0.73; 95% CI [0.13, 4.03]; *p* = 0.71) compared to moderate fluid resuscitation; however, the difference was not statistically significant (Fig. 3). None of the studies reported complete data for low-, moderate-, and high-resuscitation fluids. Moreover, only Cuéllar-Monterrubio et al. [22] compared moderate versus low; hence, a meta-analysis could not be performed.

#### Systemic or local complications

**Fig. 4:**
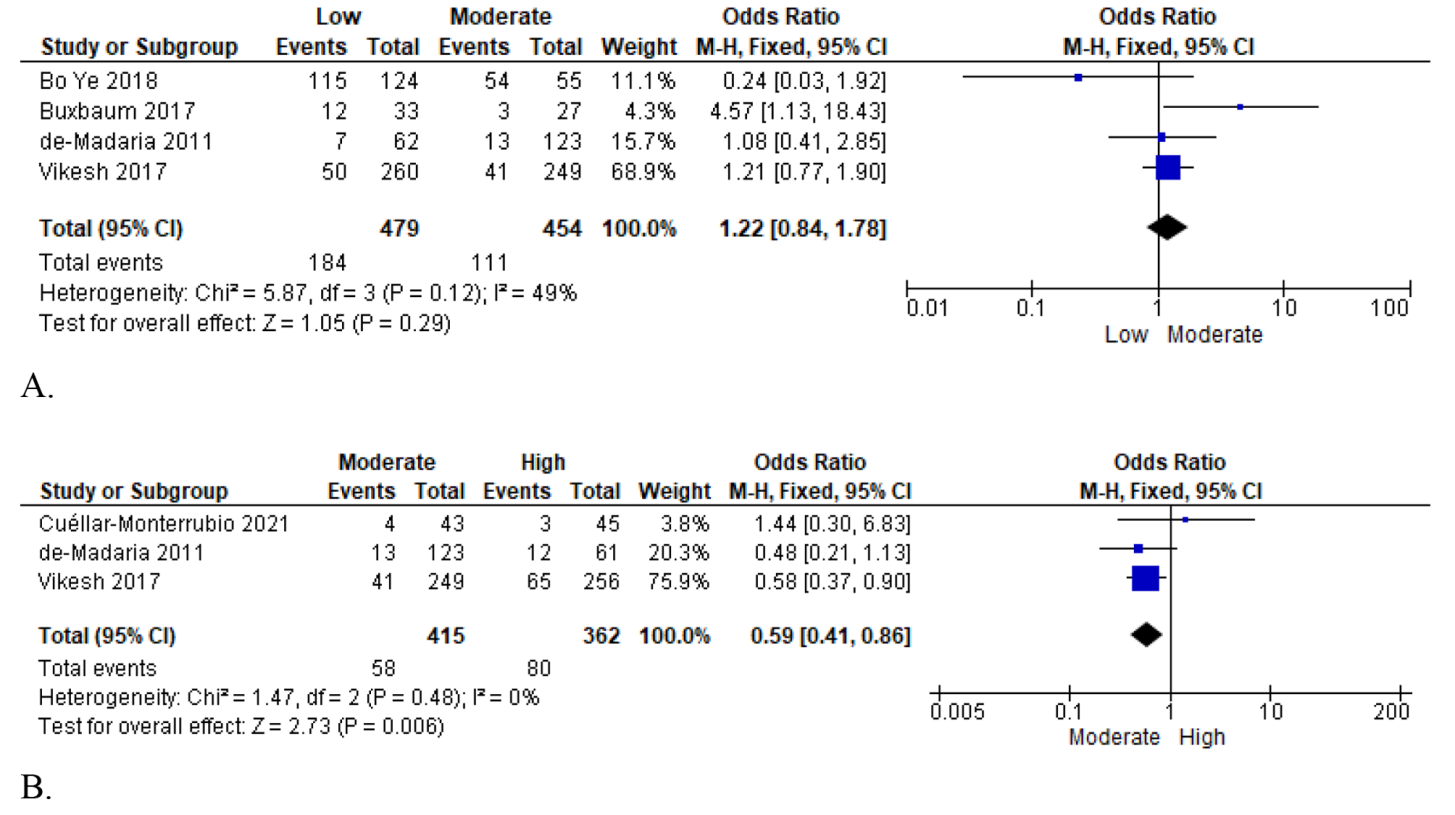
First plot of comparison: Systemic/local complications, outcome (**A**: Low vs. Moderate; **B**. Moderate vs. high)

Notable systemic or local complications associated with acute pancreatitis are acute kidney injury and heart failure. Hence, data on local complications associated with administering either high or low infusion fluid volumes were reported in five high-quality RCTs that involved 1,647 patients [18, 21–24, 26]. Four studies, including 933 participants, reported data comparing low vs. moderate fluid resuscitation [18, 21, 24, 26]. Among the studies, the heterogeneity test result (I² = 49%) was low; hence, the data were statistically analyzed using the random effects model, and the pooled meta-analysis results showed that moderate fluid therapy was correlated with improved outcomes for systemic complications (OR = 1.22; 95% CI [0.84, 1.78]; *P* = 0.29) (Fig. 4: A).

Three studies, including 1345 participants, compared moderate versus high or aggressive fluid resuscitation [21, 22, 26]. Since the studies showed no variance among them through the heterogeneity test (I² = 0%), a fixed-effect model was used for statistical analysis. The meta-analysis results showed no significant difference between moderate and aggressive (high) fluid resuscitation regarding improving systemic complication outcomes in acute pancreatitis patients (OR = 0.59; 95% CI [0.41, 0.86]; *P* = 0.006) (Fig. 4: B). A meta-analysis comparing low versus high resuscitation fluids was not conducted as no studies offered comparison data.

#### Systemic inflammatory response syndrome (SIRS development/ persistence)

**Fig. 5:**
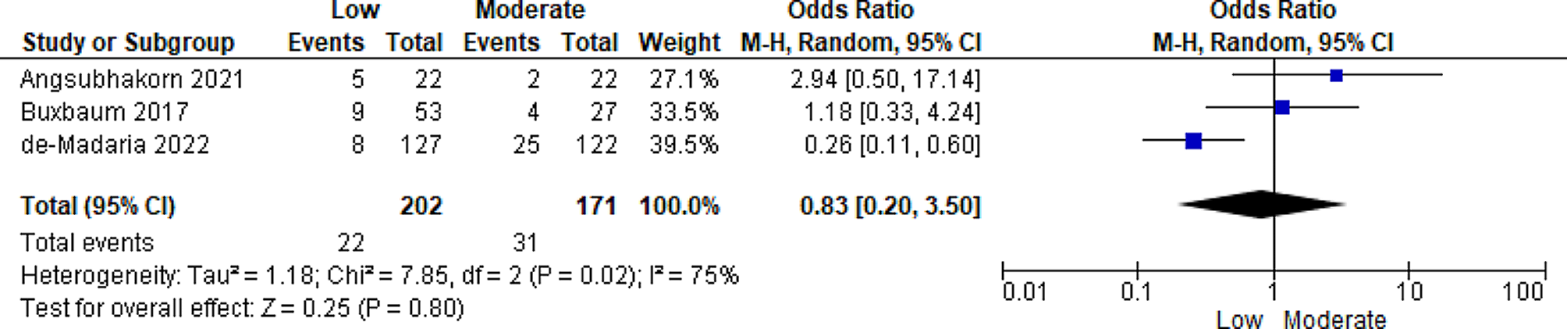
Forest plot of comparison: three SIRS (development/persistence), outcome on low vs. moderate

SIRS persistence was reported in four included studies, with a sample size of 433 participants [18–20, 22]. Cuéllar-Monterrubio et al. [22] compared non-aggressive (low) versus moderate intravenous fluid infusions. The heterogeneity test (I²) established substantive variance among the included studies (I² = 75%); thus, a random-effects model was applied in the statistical analysis. The meta-analysis results revealed by the pooled results showed a significant correlation between low fluid resuscitation and the persistence or development of SIRS. However, there was not much difference in moderate fluid resuscitation (OR = 0.83; 95% CI [0.20, 3.50]; *p* = 0.80) (Fig. 5).

#### Persistent organ failure

**Fig. 6:**
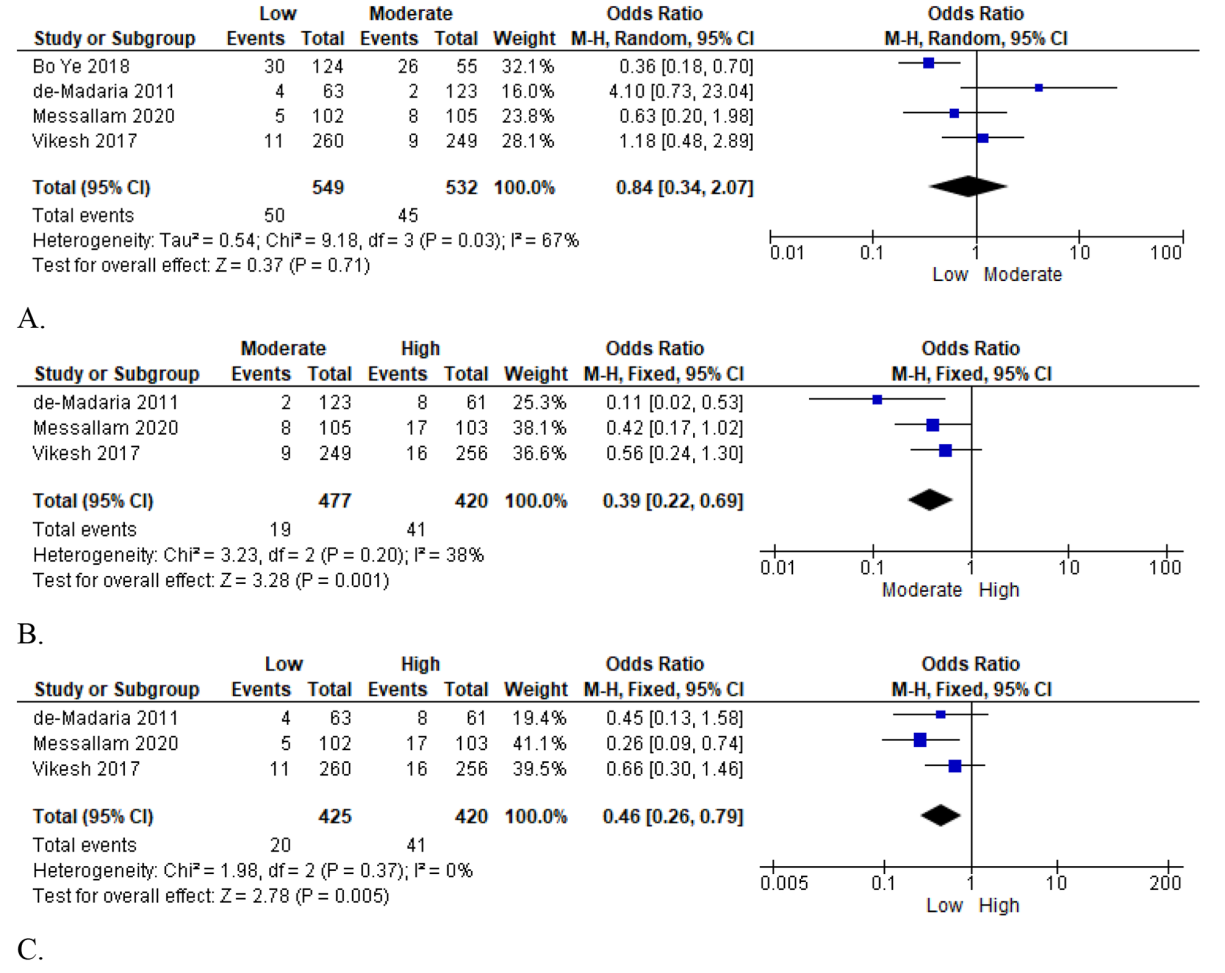
Forest plot of comparison: persistent organ failure and outcomes for (**A**: Low vs. moderate; **B**: Moderate vs. high; **C**: Low vs. high)

Persistent organ failure was reported in four included studies, including 1,746 participants, and a comparison of low vs. moderate, moderate vs. high, and low vs. high fluid resuscitation was possible [21, 23, 24, 26]. In the first meta-analysis that compared low versus moderate fluid resuscitation, the heterogeneity test indicated a significant variance between the included studies (I² = 67%); thus, the random effects model was applied in the statistical analysis. The pooled analysis results reported an association between organ failure incidence and infusion of low intravenous fluid resuscitation, implying that low resuscitation fluids increased the likelihood of organ failure in acute pancreatitis patients or did not minimize the incidence of organ failure. However, there was not much difference when compared to infusion with moderate fluids; the odds ratio was as follows: (OR = 0.84; 95% CI [0.34, 2.07]; *p* = 0.71); (Fig. 6: A).

Similarly, three studies compared moderate and high intravenous fluid infusions. Since the heterogeneity among the studies was low (I² = 38%), a fixed-effects model was adopted for the statistical analysis. Pooled meta-analysis results showed a significant association between persistent organ failure and moderate fluid resuscitation (OR = 0.39; 95% CI [0.22, 0.69]; *p* = 0.001), but the difference was not statistically significant (Fig. 6: B). The above results imply that high intravenous fluid resuscitation would likely reduce organ failure in patients with pancreatitis.

On the same note, the same studies also compared low and high fluids. In the meta-analysis, a fixed-effects model was applied to the statistical summation of the effect results since studies showed no inter-study heterogeneity (I² = 0%). The pooled results showed a strong association between organ failure persistence and infusion using low fluid resuscitation (OR = 0.46; 95% CI [0.26, 0.79], *p* = 0.005), suggesting that high fluid resuscitation improved organ failure outcomes or minimized the risks and incidences of organ failure in patients with acute pancreatitis (Fig. 6: C).

#### Mortality

**Fig. 7:**
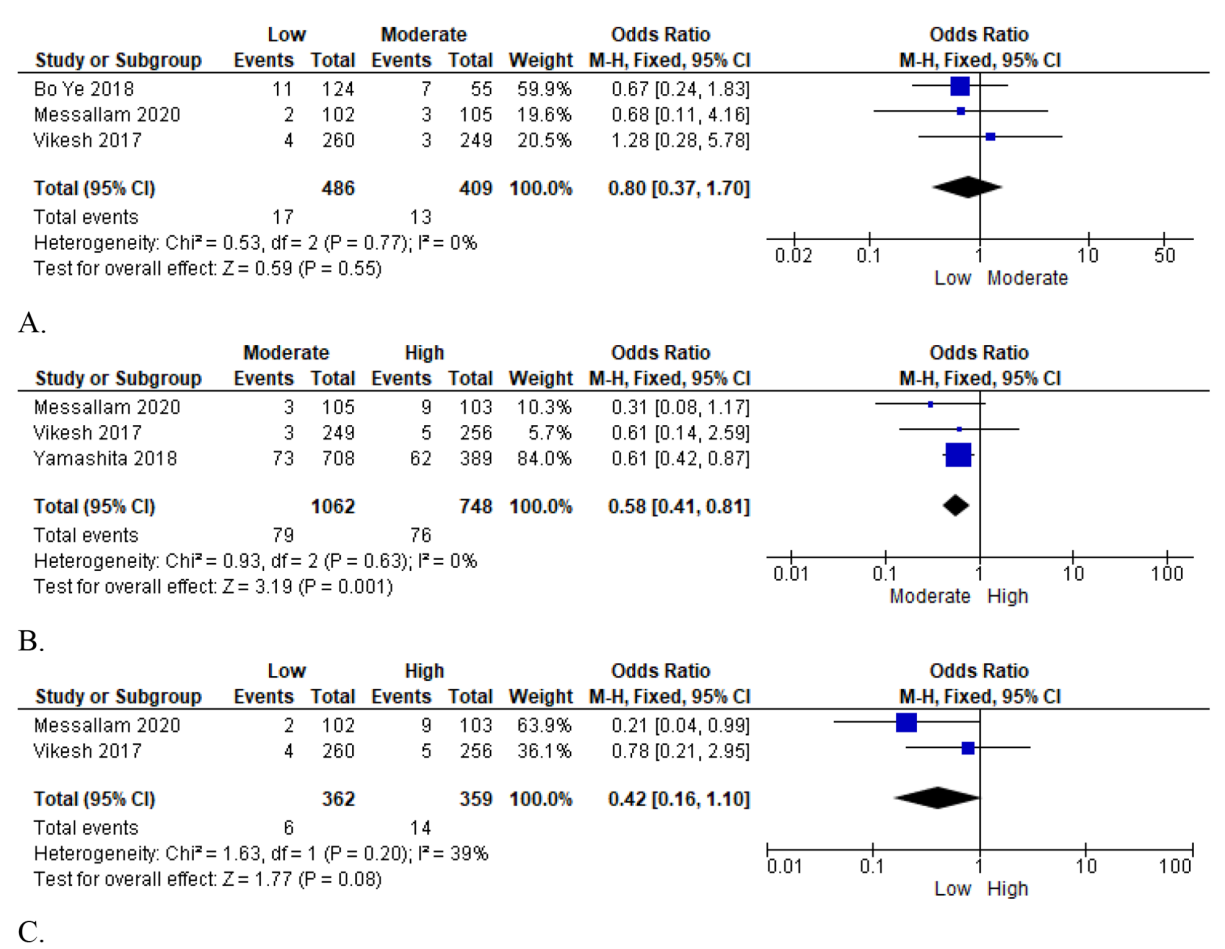
First plot of comparison: five mortality outcomes, outcomes for (**A**: Low vs. Moderate; **B**: Moderate vs. high; C: Low vs. high)

Data on the incidence of mortality were reported in four of the included studies, with a large pooled sample size of 2,596 patients who were administered high, moderate, or low fluid infusion volumes [23–26]. This study assessed the mortality outcomes of low and moderate fluid resuscitation in the first comparison. Through the inclusion of three studies with no inter-study heterogeneity (I² = 0%), the pooled results showed that infusion through low fluid resuscitation was associated with higher mortality than moderate fluid resuscitation (OR = 0.80; 95% CI [0.37, 1.70]; *p* = 0.55) (Fig. 7: A). A fixed-effects model was used to summate the overall effect results statistically. The results suggest moderate fluid resuscitation contributes to low mortality in acute pancreatitis patients.

Nevertheless, the included studies also compared moderate to high fluid resuscitation. In the fixed model, the meta-analysis results showed a higher correlation between mortality and moderate than high fluid resuscitation infusion rates (OR = 0.58; 95% CI [0.41, 0.81], *p* = 0.001), suggesting that higher fluid resuscitation led to reduced mortality risks in acute pancreatic patients. There was no inter-study heterogeneity among the assessed articles (I² = 0%) (Fig. 7: B).

Finally, a comparison between low and high fluid resuscitation volumes was performed, and statistical analysis was performed using the fixed effects model due to low inter-study heterogeneity (I² = 39%). Based on the meta-analysis results, low fluid infusion was associated with higher mortality rates than high fluid resuscitation (OR = 0.42; 95% CI [0.16, 1.10]; *p* = 0.08); hence, higher fluid resuscitation volumes were likely to yield improved or lower mortality outcomes in patients with acute pancreatitis (Fig. 7: C).

#### Outcomes and results: comparison by infusion volume types

**Fig. 8:**
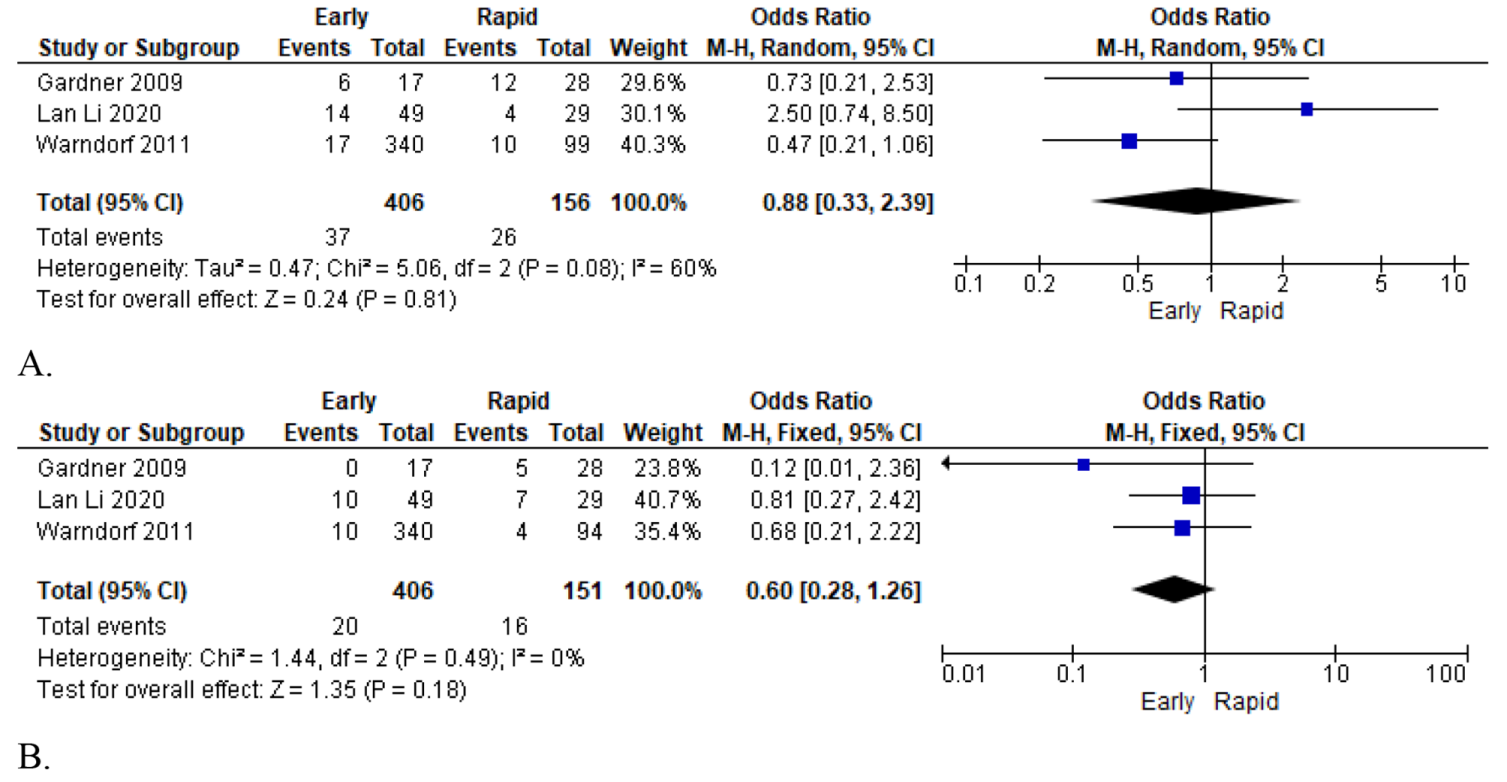
Forest plot of comparison: six outcomes by volume type (early vs. rapid), outcomes for (**A**: Organ failure; **B**: Mortality)

Three included studies reported data comparing studies based on infusion volume types: early versus rapid (late) fluid resuscitation [27–29]. Early resuscitation was defined as receiving ≥ 1/3 of the total 72 h fluid volume within 24 h of presentation. In contrast, late resuscitation was defined as receiving ≤ 1/3 of the total 72 h fluid volume within 24 h of presentation. Three high-quality studies with a large population sample of 1,391 particulates were included, and the main outcomes assessed were organ failure and mortality. With respect to organ failure, the random-effects model was applied in the meta-analysis because the heterogeneity test revealed no significant variance among the included studies (I² = 60%). The pooled analysis results showed that early fluid resuscitation reduced the rates of organ failure compared with rapid fluid resuscitation (OR = 0.88; 95% CI [0.33, 2.39]; *p* = 0.81), suggesting that rapid fluid infusion was attributed to increased organ failure in patients with AP (Fig. 8: A).

A fixed-effect model was applied in the meta-analysis for mortality outcomes because there was no inter-study heterogeneity (I² = 0%). Pooled statistical results showed a strong association between early fluid resuscitation and reduced mortality risk (OR = 0.60; 95% CI [0.28, 1.26]; *P* = 0.18), implying that rapid fluid resuscitation volumes were likely to increase mortality in patients with AP (Fig. 8. B).

#### Study publication bias

**Fig. 9:**
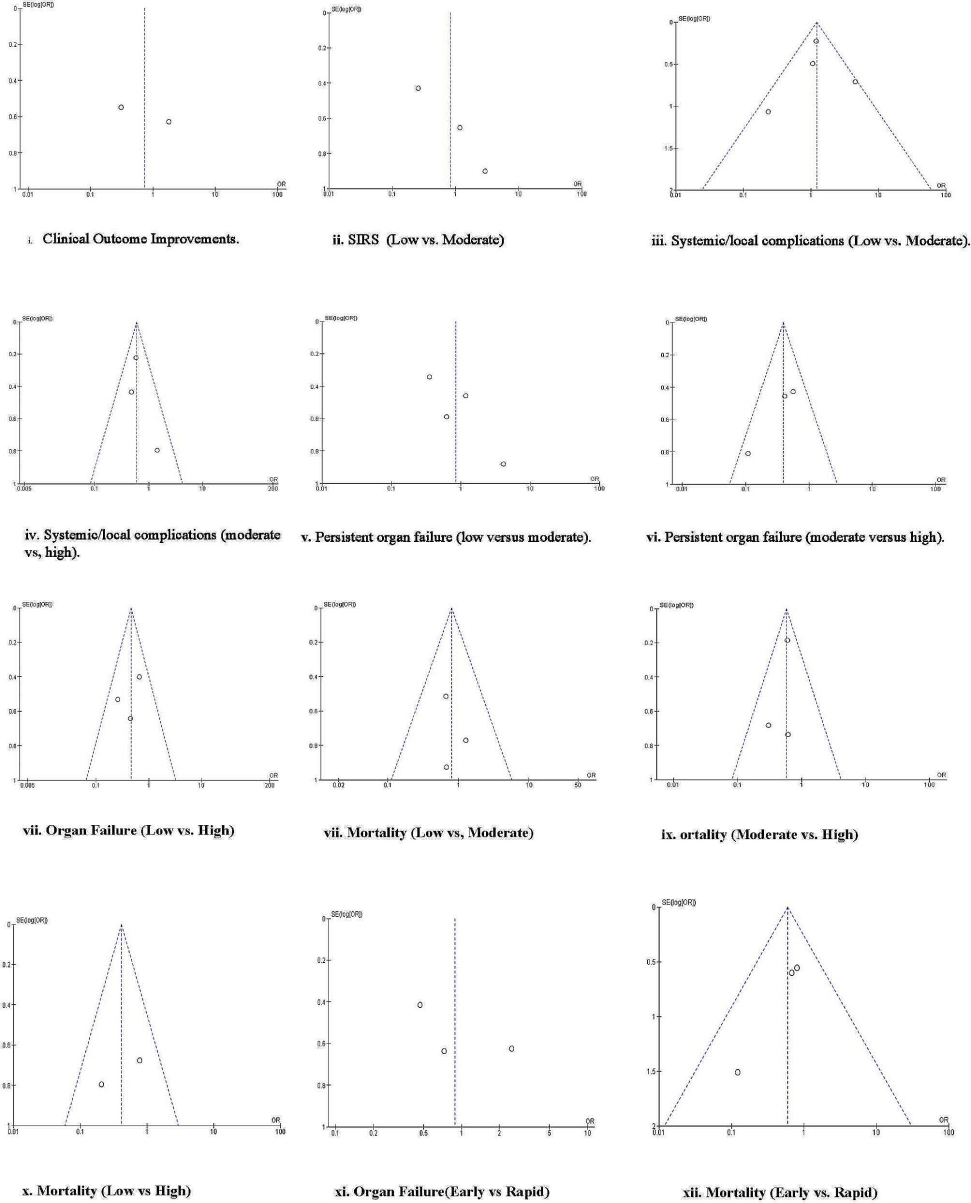
Funnel plot analysis

The results of funnel plot analysis are shown in Fig. 9. Potential publication bias was based on visual analyses of funnel plots. The distribution of (I, II, III, V, VIII, XI) was symmetrical, suggesting no evidence of publication bias. However, the funnel plots for (IV, VI, VII, IX, X, and XII) were asymmetrical, suggesting a potential publication bias.

## Discussion

### Summary of findings

This study evaluated the efficacy of intravenous fluid resuscitation on the clinical outcomes of patients with acute pancreatitis. In particular, this study sought to ascertain the best choice of clinical intervention between the various fluid infusion resuscitation rates recommended by physicians in treating acute pancreatitis. Hence, low versus moderate versus high infusion rates were compared regarding crucial clinical outcomes, including systemic inflammatory response, clinical improvement outcomes, systemic complications, mortality risks, and persistent organ failure. The study results showed that moderate fluid resuscitation showed better outcomes regarding clinical improvements, systemic complications, minimizing SIRS persistence, the persistence of organ failure, and reduced mortality rates, and similar results were observed in favor of high intravenous fluid volumes. Contrary results were observed for overall clinical improvement, where moderate fluid infusion rates presented superior clinical outcomes to higher-rate fluid resuscitation. Higher risks were associated with administering low fluid infusion volumes for systemic or local complications. This implies that moderate or high fluid resuscitation was attributed to fewer systemic complications in patients with severe pancreatitis. Finally, early fluid resuscitation improved the associated organ failure outcomes and mortality rates compared to rapid or late resuscitation.

### Correlation with previous literature findings

Consistent with our findings, previous studies have also reported similar observations. For instance, Laplante et al. [30] showed that higher infusion fluid volumes and rates led to an enhanced systemic inflammatory response in the organs. According to previous studies, the pathophysiology of acute pancreatitis relies on the activation of trypsinogen and an increased inflammatory response, leading to adverse events and organ failure. Therefore, administering high-rate infusion fluid volumes improves the systemic inflammatory response and prevents organ failure. This implies that administering a higher fluid resuscitation volume would minimize the adverse risks of SIRS in patients with acute pancreatitis. Studies have also shown that higher-rate fluid infusion is associated with clinical improvement. In supporting evidence that compared higher and moderate infusion rates for clinical improvement, Szabo et al. [11] contended that improved clinical outcomes were not associated with moderate fluid resuscitation in acute pancreatitis, acknowledging the superiority of higher infusion rates. Furthermore, Szabo et al. ascertained that high-rate fluid resuscitation improved clinical outcomes in acute pancreatitis, especially in pediatric patients [11].

This systematic review and meta-analysis also noted an increased incidence of mortality among critically ill patients treated with intravenous fluid resuscitation. Based on these results, a higher fluid resuscitation infusion did not significantly reduce mortality rates compared to low intravenous fluid resuscitation. This indicates that both low and high fluid volumes are associated with mortality. A meta-analysis by Gad et al. [31] contributed to the recent findings by concluding that aggressive intravenous fluid therapy given in high infusion volumes did not reduce mortality risks but increased the risks of pulmonary edema and acute kidney injury. Crosignani et al. [3] also agreed with the outcomes of the present meta-analysis by asserting high mortality rates in low-fluid (5–10 ml/kg/h) resuscitation in acute pancreatitis [3]. Similar observations were noted in other studies by Brown et al. [32] and Aggarwal et al. [33], who reported high death rates in low fluid resuscitation volumes with hemoconcentration. The authors argued that low fluid levels lead to insufficient blood supply and flow in the pancreas, leading to pancreatic necrosis and, eventually, death [32, 33]. Furthermore, Wall et al. suggested that high fluid resuscitation in acute pancreatitis is associated with higher mortality than low fluid resuscitation [34].

Concerning findings on local or systemic complications, Sweeney et al. [35] associated moderate fluid infusion with reduced systemic complications compared to low fluid resuscitation rates. In another study, Ocskay et al. [36] reported that moderate fluid resuscitation reduced local complications in acute pancreatitis. Finally, regarding the length of hospital stay, previous findings resonate with the present findings. Casey et al. [37] supported the findings that moderate fluid resuscitation in acute pancreatitis reduces patients’ lengths of stay in the hospital. Studies by Di Martino et al. [38] and Leppäniemi et al. [4] noted that moderate fluid resuscitation reduces the length of hospital stay in acute pancreatitis.

#### Limitations

We were unable to assess the effect of the type of intervention fluid on the study outcomes due to lack of comparative studies. Half of the included studies recruited patients who had received any type of intravenous resuscitation, one fourth exclusively used Lactated Ringer’s solution. Although two studies classified patients who had received saline (normal or D5) or Ringer’s lactate solution, the [23, 27], patient outcomes based on the type of fluid administered was not reported. The use of balanced crystalloids in fluid therapy in critically ill patients has been associated with lower mortality rates compared to normal saline [39].

Furthermore, over half of the included studies had attrition, reporting or other biases which may have contributed to high heterogeneity between studies for clinical outcomes, SIRS persistence, and organ failure persistence.

## Conclusions

Even though physicians continue to recommend low-, moderate-, and high-rate fluid resuscitation to patients, the study concluded that high fluid infusion rates accorded the best clinical outcomes for patients with pancreatitis. High versus low rates were associated with reduced SIRS risk incidence and shortened hospital length of stay among the patients. Low certainty evidence also supported moderate fluid administration as a starting point for pancreatitis patients with initial manifestations.

Generally, owing to the lack of robustness of studies that vividly and analytically provided a uniform comparison between low vs. moderate vs. high fluid therapy, this study recommends further research, including studies with high methodological quality, to validate the findings of this study. Specifically, there is a need for studies reporting clinical outcomes with low, moderate, and high fluid infusion rates in patients with different AP severity to assess any potential relationship between fluid infusion rate and disease severity. In addition, future studies investigating the effect of different types of fluids in AP are required.

## Data Availability

The datasets used and/or analyzed during the current study are available from the corresponding author on reasonable request.
